# CathROB: A Highly Compact and Versatile Remote Catheter Navigation System

**DOI:** 10.1155/2017/2712453

**Published:** 2017-05-25

**Authors:** Laura Cercenelli, Barbara Bortolani, Emanuela Marcelli

**Affiliations:** Laboratory of Bioengineering, Department of Experimental Diagnostic and Specialty Medicine, University of Bologna, Bologna, Italy

## Abstract

Several remote catheter navigation systems have been developed and are now commercially available. However, these systems typically require specialized catheters or equipment, as well as time-consuming operations for the system set-up. In this paper, we present CathROB, a highly compact and versatile robotic system for remote navigation of standard tip-steerable electrophysiology (EP) catheters. Key features of CathROB include an extremely compact design that minimizes encumbrance and time for system set-up in a standard cath lab, a force-sensing mechanism, an intuitive command interface, and functions for automatic catheter navigation and repositioning. We report in vitro and in vivo animal evaluation of CathROB. In vitro results showed good accuracy in remote catheter navigation and automatic repositioning (1.5 ± 0.6 mm for the left-side targets, 1.7 ± 0.4 mm for the right-side targets). Adequate tissue contact was achieved with remote navigation in vivo. There were no adverse events, including absence of cardiac perforation or cardiac damage, indicative of the safety profile of CathROB. Although further preclinical and clinical studies are required, the presented CathROB system seems to be a promising solution for an affordable and easy-to-use remote catheter navigation.

## 1. Introduction

In the last decade, robotics has expanded significantly in the field of minimally invasive cardiology, especially for transcatheter radiofrequency (RF) ablation procedures to treat complex arrhythmias such as atrial fibrillation [1-3]. The main advantages of robotic remote catheter navigation include improved catheter stability and reduced total radiation exposure to both the patient and the operator.

Today, two principal technologies for remote catheter navigation are available. One utilizes magnetic field vectors to navigate proprietary sensorized catheters, the other uses electromechanical elements to robotically maneuver standard electrophysiology (EP) catheters or dedicated sheaths where standard catheters are inserted. Based on these two technologies, four remote catheter navigation systems are now commercially available (Table 1).

The Niobe (Stereotaxis Inc., MO, USA) is a magnetically driven system that uses magnetic fields generated by two external large magnets located on either side of the patient to move and navigate with three degrees of freedom (3-DOF), a specially designed catheter that includes magnets in its distal tip [4–6]. This magnetic catheter is soft without an excessive contact force; thus, it potentially reduces the risk of cardiac perforation; however, lower forces applied when using this catheter may result in fewer transmural (effective) ablation lesions [7]. Some concerns with the Niobe system are related to the need for a room dedicated to the magnets and the complexity of the overall system set-up [1, 3, 7–9].

The Sensei robotic navigation system (Hansen Medical Inc., CA, USA) includes custom-designed steerable sheaths where standard EP catheters are inserted to allow their remote manipulation using a 3-DOF joystick [10, 11]. Despite this system providing superior catheter stability with respect to manual procedure, mechanical complications are possible due to the rigidity of the custom-designed sheaths entering the patient [3]. Recently, the driving components of the Sensei system have also been adapted for navigation of endovascular catheters, leading to the Magellan robotic system [12–15].

The Amigo remote catheter system (Catheter Precision Inc., NJ, USA) is a robotic arm attached to the patient's table which allows 3-DOF manipulation of standard tip-steerable EP catheters, using a remote control handle that mimics the standard catheter handle [16, 17]. From clinical experience, the Amigo system seems to be safe and effective, although it remains quite cumbersome in the cath lab (size: 101 × 137 × 112 cm; weight: 32 kg) [18].

The CorPath vascular robotic system (Corindus Vascular Robotics, MA, USA) comprises a remote manipulation unit and a bedside unit (composed of a specially designed single-use cassette mounted on a robotic drive) for maneuvering coronary guidewires and balloon/stent devices during percutaneous coronary interventions [19, 20].

The major obstacles to a wider adoption of these robotic remote catheter navigation systems remain their complexity and high cost [6, 7], as well as the longer procedure times they generally require as compared to the manual procedure, mainly due to the time-consuming operations for system set-up [1, 3, 7–9].

In the recent years, several research groups have proposed master–slave systems for remote catheter navigation with 2-DOF [21–23] or 3-DOF [24, 25] and with incorporated force feedback [21, 22, 25], but they are still in the early phases of implementations.

We have previously presented a prototype of a telerobotic system to remotely manipulate standard steerable EP catheters from a suitably shielded room [26, 27]. In the present study, we introduce CathROB, an advanced prototype for remote catheter navigation that we developed in cooperation with Tre Esse Progettazione Biomedica s.r.l. [28]. The system is designed to minimize complexity, encumbrance, and time for system set-up in a standard cath lab.

In this paper, we describe the CathROB system design and in detail its major components and features; furthermore, we report our in vitro and in vivo animal evaluations of CathROB in performing a safe remote catheter navigation and RF ablation.

## 2. Materials and Methods

### 2.1. CathROB Description

**Figure 1:**
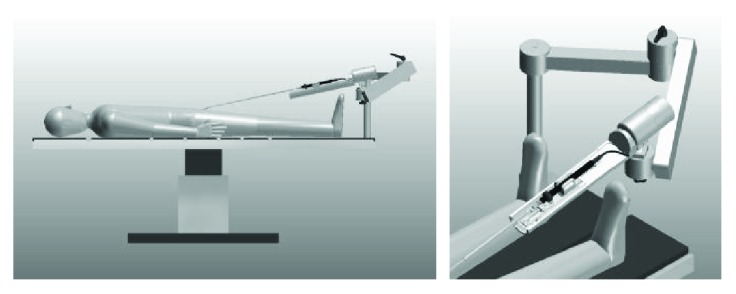
3D rendering of the CathROB system attached to a standard cath lab table via an articulated arm.

CathROB is a remotely controllable electromechanical device designed to navigate conventional tip-steerable EP catheters. The idea is to provide a practical and compact robotic manipulator, which replicates remotely the manual catheter handling performed by the interventional cardiologist at bedside. A 3D rendering of the CathROB system design is illustrated in Figure 1.

The key benefit of CathROB over the existing systems is its compact and lightweight design that allows the fast and easy installation in a cath lab (Supplementary Video 1 available online at https://doi.org/10.1155/2017/2712453).

The system is intrinsically safe, since it is designed to manipulate standard catheters inserted through conventional introducer sheaths, without requiring special devices entering the patient. Therefore, the system does not change the normal catheter forces within the heart, and the catheter retains all of its normal bending and buckling properties, just like it is manipulated by the operator's hand.

**Figure 2:**
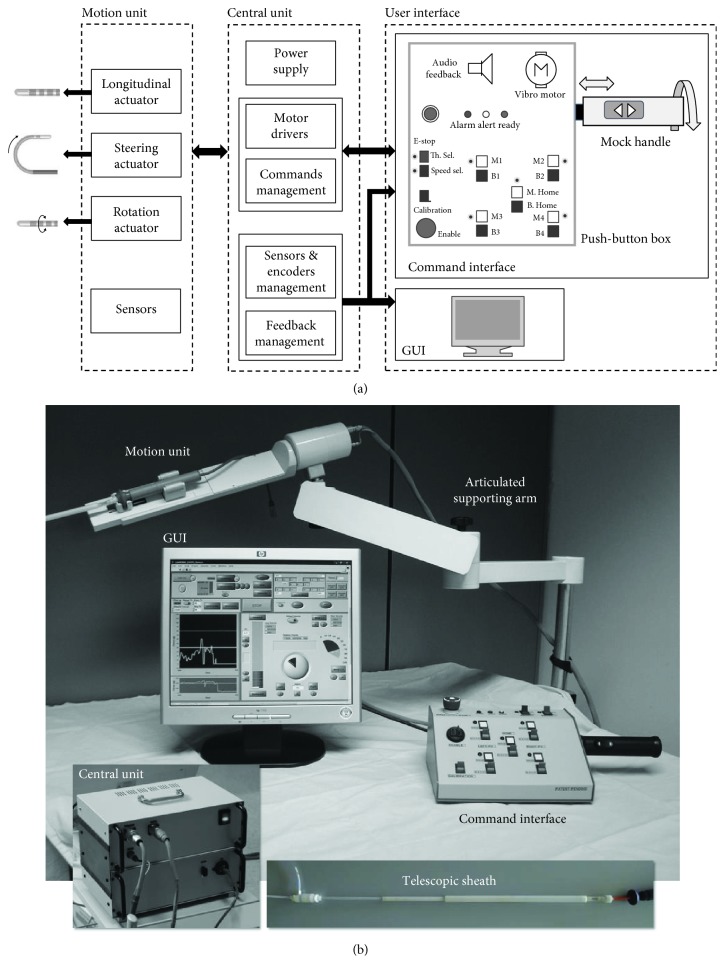
Scheme of CathROB architecture (a) and photograph of the overall system (b).

The CathROB mainly consists of a *Motion Unit*, a *Central Unit*, and a remote *User Interface* composed of both a command interface and a graphical user interface (GUI) (Figure 2).

The robotic manipulator is also equipped with a proximal force-sensing mechanism to make the operator aware of the resistance encountered by the catheter while advancing, which allows an additional safety control to avoid the risk of cardiac tissue perforation while remotely navigating the catheter.

A detailed description of the system is presented in the following paragraphs.

#### 2.1.1. Motion Unit and Supporting Arm

**Figure 3:**
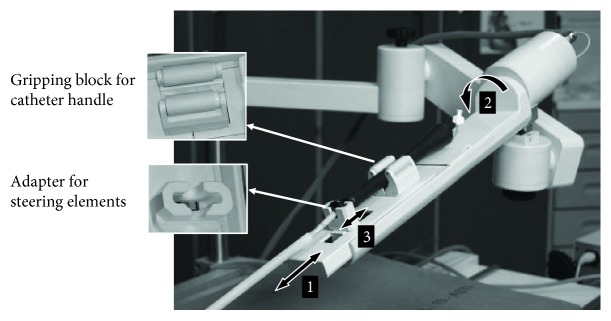
CathROB Motion Unit that controls catheter movements in 3-DOF. 1: longitudinal (advance/withdraw); 2: rotational (clockwise/counter clockwise); 3: tip steering (bend/straighten).

The *Motion Unit* includes three controllable actuators composed of DC micromotors with gearboxes (Portescap, PA, USA) and rotary optical encoders (Bourns, CA, USA) used to count and track the catheter movements. The unit allows navigation of the catheter into 3-DOF: (1) longitudinal movement (advance/withdraw), (2) rotational movement (clockwise/counter clockwise rotation), and (3) tip-steering movement (bend/straighten) (Figure 3). For each DOF, the motor speed can be adjusted within the following ranges: 13 to 18 mm/s, 30 to 45 degrees/s, and 2 to 4 mm/s.

**Figure 4:**
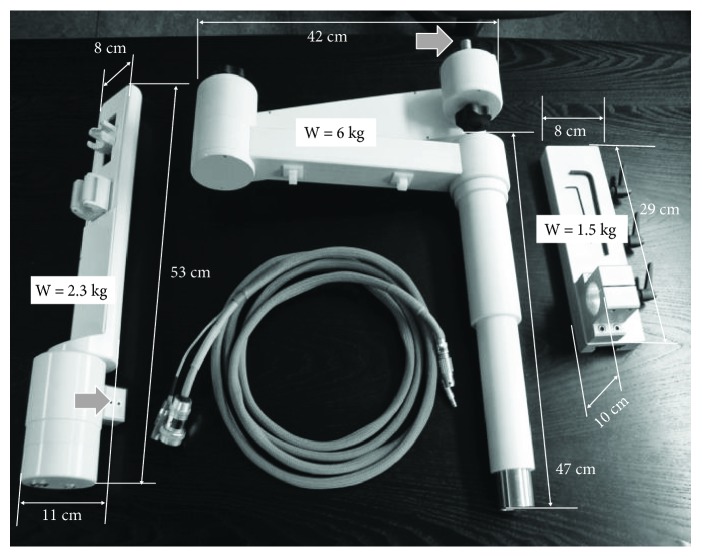
Weight and size of the CathROB Motion Unit (left) and of the supporting arm (centre) that includes the anchoring plate for the attachment to the cath lab table (right).

The *Motion Unit* has a minimum size of 53 × 8 × 110 cm for the complete withdrawal position, and a maximum size of 73 × 8 × 11 cm for the maximal longitudinal extension position. It weighs only 2.3 kg (Figure 4).

The CathROB system can be rapidly attached to a standard cath lab table via an articulated supporting arm (Figures 3 and 4). The arm (overall weight 7.5 kg) is composed of two adjustable articulations (each one with an overall size of 42 × 8 × 8 cm) hinged on a vertical cylindrical shaft (L = 47 cm, Ø = 8 cm) that can be attached to the rail of a cath lab table using a specifically designed anchoring plate (Figure 4). The articulated supporting arm allows to easily adjust the height, the lateral position, the roll, and jawing angles of the manipulator with respect to the patient (Supplementary Video 1).

The *Motion Unit* can be attached to the supporting arm via an interlocking mechanism between a drilled block in the lower part of the *Motion Unit* and a shaft protruding from the last joint of the articulated arm (see grey arrows in Figure 4()).

The catheter handle is held in place on the *Motion Unit* via a gripping block made of two jaws pretensioned by a spring and ending with two silicone-coated rollers that ensure a stable grip for different geometrical handles (Figure 3). Before mounting the catheter on the robotic hand, it is passed through a rigid *telescopic sheath* made of Delrin that helps to stiffen and stabilize the connection between the robotically controlled catheter handle and the standard introducer sheath at the venous access (Figure 2(b)). The distal tip of the *telescopic sheath* attaches to the hub of the standard introducer sheath used for venous access; therefore, no part of the *telescopic sheath* enters the patient's body. The *telescopic sheath* has been specifically designed to reduce the frictional forces while moving the catheter inside it, as well as to reduce the friction between the catheter and the inner wall of the standard introducer sheath entering the patient [29].

**Figure 5:**
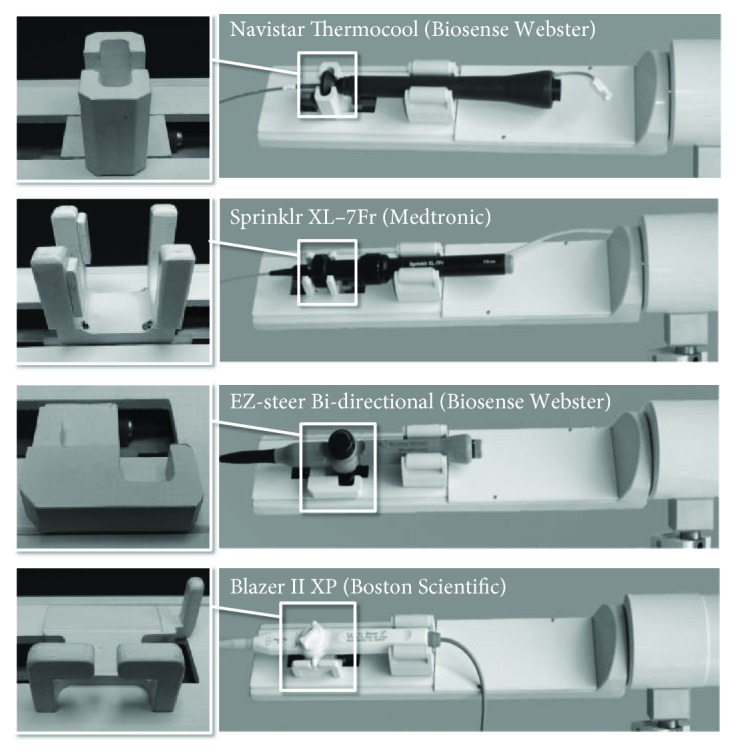
CathROB adaptation to manipulate various models of commercially available tip-steerable EP catheters.

CathROB is conceived to be an open platform, which can accommodate many commercially available EP catheters. Adapters for four different commercial catheter models are currently provided (Figure 5). These models are all tip-steerable EP/ablation catheters, with different elements in the hand piece to control the tip deflection. The Navistar Thermocool (Biosense Webster Inc.) and the Sprinklr XL-7Fr (Medtronic Inc.) have a thumb knob that controls the travel of a piston used for tip steering: deflection when the thumb knob is pushed forward, straightening when the thumb knob is pulled back. The EZ-steer Bi-directional (Biosense Webster Inc.) has a rocker lever in the handle, which is used to deflect the tip with two 180° opposed single plane curves, with various combinations of symmetrical/asymmetrical curves. Blazer II XP (Boston Scientific) is another model of bidirectionally steerable catheter with a thumb-actuated biwing steering knob in the handle that can provide both symmetric and asymmetric curve configurations.

Each adapter can be quickly coupled to the *Motion Unit* and is designed to be disposable or sterilizable.

Both the gripping block for the catheter handle and the adapters for the steering elements are designed to ensure a fast manual plug-in and removal of the catheter handle from the *Motion Unit*, thus allowing easy switching from robotic to manual catheter handling (Supplementary Video 1).

#### 2.1.2. Central Unit and User Interface

**Figure 6:**
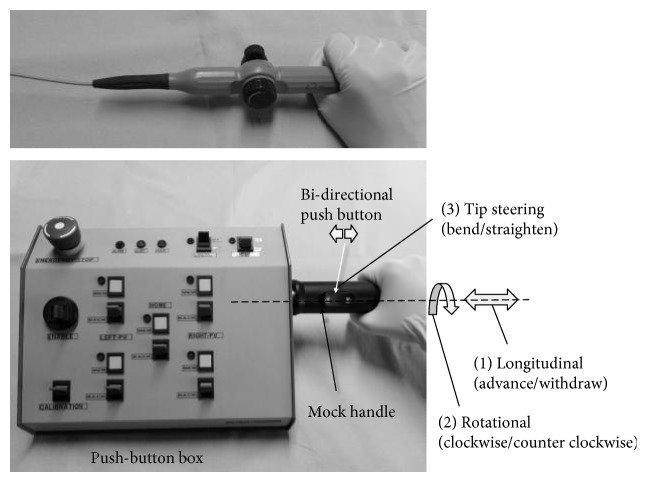
The new intuitive CathROB command interface (bottom) composed of a mock handle that mimics a standard catheter handle (top) and a push-button box.

The *Central Unit* mainly comprises power supply circuitry and a programmable controller (CompactRIO, National Instruments, TX, USA) programmed in LabVIEW 2014 (National Instruments, Austin, TX, USA). The *User Interface* consists of a command interface and a GUI. The control joystick, previously developed for the initial prototype [28], has been replaced with a more intuitive command interface, which is composed of a mock handle that mimics the conventional EP catheter handle maneuvered manually by physicians, and a push-button box (Figure 6()). The longitudinal movement (advance/withdraw) is controlled by pushing/pulling the mock catheter handle anchored to the push-button box; the rotational movement is controlled by rotating clockwise/counter clockwise the mock handle around its axis; the steering of the catheter tip is controlled by pushing a bidirectional button on the mock handle (Figure 6()).

The push-button box is equipped with buttons used to save a reference navigation position (Home) and to start the automatic catheter navigation to preloaded navigation sites. During remote navigation, up to four endocardial target positions can be saved and used as targets for the automatic catheter repositioning.

**Figure 7:**
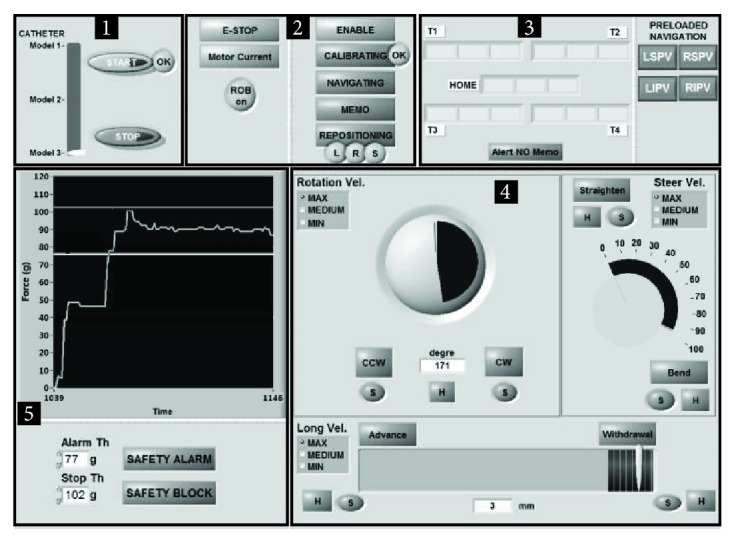
The CathROB GUI detailed in Table 2.

The *GUI*, developed using the graphical programming language LabVIEW 2014 (National Instruments), has been divided into 5 functional blocks (Figure 7), including softkeys and indicators that are described in detail in Table 2.

#### 2.1.3. Automatic Catheter Repositioning Algorithm

The system is provided with an automatic catheter navigation and repositioning algorithm to guide the catheter to preselected and memorized endocardial targets.

The algorithm for automatic navigation has been designed in order to ensure a safe combination of catheter movements in 3-DOF.

First of all, the catheter tip is straightened, simultaneously retracted until the tip is completely straightened. In this “safe” catheter configuration (i.e., no bending and no contact with the cardiac wall), the catheter is automatically rotated to the rotational target position. Finally, the bending of the tip and the advancement/retraction of the catheter are carried out simultaneously to reach the steering and longitudinal target coordinates.

#### 2.1.4. Force-Sensing Mechanism

The force-sensing mechanism is based on the use of a piezoresistive force sensor (MICRO SWITCH Force Sensor, FS Series, Honeywell, MN, USA) inserted among the mechanical elements used to transmit the advancement/withdrawal movement in the *Motion Unit*. A compression spring is used to preload the force sensor in order to absorb all forces due to oscillations of the *Motion Unit*, which are not directly related to the catheter advancing.

Although this sensing mechanism reads the force from the robotic hand rather than from the catheter tip, it can provide indication of the catheter contact force with the endocardium, since the rigid *telescopic sheath* avoids the catheter deflection between the handle and the venous access and ensures transmission of the distal tip contact force to the proximal force sensor [27].

Two force level thresholds are provided: a first “alarm threshold,” which is used to alert the operator that the catheter-endocardium contact is achieved, and a higher “stop threshold,” which is used to control the automatic stop of all catheter movements, to avoid any excessive pushing force on the endocardium.

To define alarm and stop thresholds, we first performed in vitro tests to measure the catheter tip force required for mechanical perforation (Perforation Force, PF) of cardiac tissue of an excised ovine heart. For the test, we anchored the tip of a standard commercial ablation catheter (EZ-steer Bi-directional, Biosense Webster) to the extension rod of a digital force gauge (Compact Force Gauge 100N, Mecmesin) in order to make the catheter integral with the gauge. This also allows to stiffen the catheter lead, so as to recreate the most critical condition of maximum peak load transferred from tip to tissue. Then, a piece of myocardial tissue, particularly the thinner regions of the right atrial wall, was pressed against the catheter tip, until achieving tissue perforation. While pressing the tissue against the catheter tip, a progressively increasing force was measured by the force gauge, until arriving at maximum force value (PF), just before the abrupt fall to zero, due to tissue perforation. Starting from the mean PF (128 ± 12 gF) obtained by averaging results of three repeated tests, the percentage for the two thresholds (60% PF for the alarm threshold, 80% PF for the stop threshold) were defined on the basis of the feedbacks received from three experienced electrophysiologists who used CathROB during the in vivo animal experiments.

To give a haptic force feedback while the catheter is remotely advanced, a vibration motor is inserted in the push-button box in the proximity of the mock handle. The vibration is activated when alarm or stop threshold is exceeded, thus providing a reaction force back to the operator's hand. An acoustic alarm is also added to the vibrational haptic feedback.

### 2.2. CathROB Evaluation

#### 2.2.1. In Vitro Evaluation

**Figure 8:**
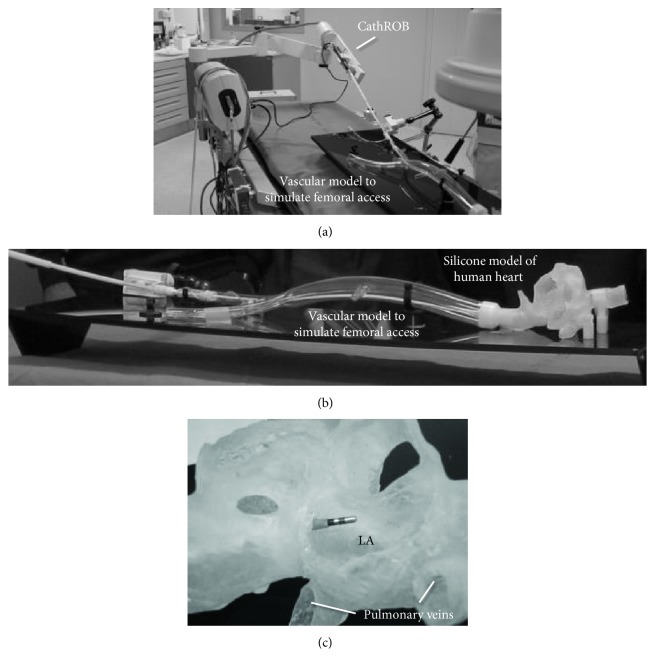
The experimental set-up for in vitro testing including (a) a rigid plastic vascular model to simulate the femoral access for the catheter and (b) a silicone model of human heart. (c) LA: left atrium.

Preliminary in vitro evaluation of CathROB was performed on a mock-up system that reproduces the cardiovascular structures in which a standard ablation catheter is navigated. The mock-up consists of a rigid plastic vascular model (Figures 8(a) and 8(b)) used to simulate the femoral access for the catheter and a silicone model of a human heart, which reproduces the right atrium (RA) and the left atrium (LA) with parts of the pulmonary veins (Figure 8(c)).

A narrow hole through the interatrial septum was created in the silicone model in order to allow the transseptal passage of the catheter to the left cardiac side. A standard 7.5 Fr ablation catheter (Navistar Thermocool 4 mm, Biosense Webster, CA, USA) compatible with the 3D mapping CARTOMERGE™ system (Biosense Webster, CA, USA) was inserted in a standard long introducer sheath and in the CathROB telescopic sheath, then it was manually advanced to the entrance of LA in the silicone model. The catheter handle was mounted on the CathROB system and the physician performed remotely the navigation and the electroanatomic mapping of the LA chamber in the silicone model.

The CARTOMERGE software was used to calculate the overall average accuracy of integration of the obtained CARTO map with the computer tomography- (CT-) derived reconstruction of the LA (i.e., “deviation index” between the CARTO map and the CT reconstruction).

Moreover, the accuracy of the automatic catheter repositioning algorithm was tested: the catheter was remotely navigated to four LA endocardial sites in the proximity of each pulmonary vein ostium that were saved as target positions using the push buttons in the CathROB command interface. For each target position, three consecutive automatic repositioning of the catheter to the saved target were repeated (resetting each time the starting position of the catheter via the automatic returning to Home). Repositioning errors were estimated from the difference, among repeated repositioning, of the tip catheter position in the CARTO map.

#### 2.2.2. In Vivo Animal Evaluation

Four sheep (49 ± 4 kg) were used for the evaluation. The experimental endpoint was to demonstrate the feasibility and safety of performing remotely the cardiac mapping and RF ablation, using the CathROB system.

In vivo experiments were carried out in a dedicated facility (the Laboratory of Preclinical and Surgical Studies, Rizzoli Orthopaedic Institute, Bologna) equipped with a fluoroscope and a CARTO system (Biosense Webster, CA, USA) for electroanatomical cardiac mapping. The experiments were carried out with the support of dedicated veterinary and medical staff. Tests were performed following a specific protocol approved by the local institutional animal care and use committee. All institutional and national guidelines for the care and use of experimental animals were followed.

**Figure 9:**
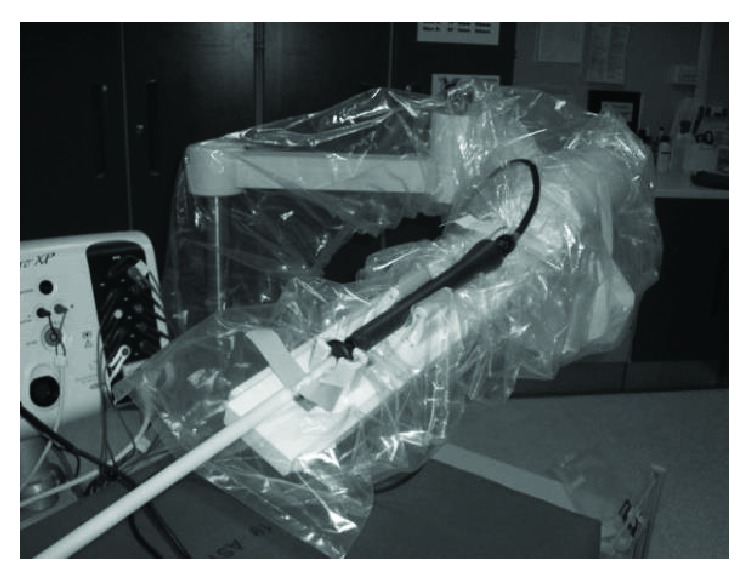
CathROB preparation for in vivo animal evaluation.

Animals were premedicated with 10 mg/kg ketamine through an intramuscular injection and placed on a surgical table. After induction of anaesthesia with 5–10 mg/kg thiopental sodium, the animals were mechanically ventilated at a tidal volume of 15 ml/kg and a respiratory rate of 20 breaths per minute with 2% fluothane and O_2_, N_2_O mixed gas. Using a standard surgical procedure, the operator performed incisions in the animal's skin and the right femoral venous access in a standard way. A long introducer sheath (Preface braided guiding sheath 77 cm multipurpose, Biosense Webster, CA, USA) was placed in the femoral vein. A standard 7.5 Fr ablation catheter (Navistar Thermocool 4 mm, Biosense Webster, USA) compatible with the 3D mapping CARTO system was preliminarily passed through the CathROB *telescopic sheath* and then it was manually advanced to the entrance of RA under direct fluoroscopy visualization. A standard diagnostic decapolar catheter (Polaris X, Boston Scientific, MA, USA) was also inserted and positioned in the coronary sinus for anatomic reference. The CathROB robotic hand was prepared for use in the sterile operating theathre by applying a disposable sterile polyethylene covering on both the *Motion Unit* and the supporting arm. The ablation catheter handle was mounted on the CathROB system via the gripping block and the proper steering adapter (Figure 9). Then, the operator manipulated the ablation catheter via the robotic hand, using the remote command interface from outside the radiation field. The RA mapping was performed remotely using the CathROB device in conjunction with the CARTO system and standard fluoroscopy. After map reconstruction, the ablation catheter was remotely navigated within the map. During catheter navigation, the switching from remote to manual control was also experimented. Finally, RF ablation was applied remotely via CathROB on two selected RA target sites (RA isthmus and RA posterior wall), while maintaining a stable catheter tip-endocardium contact, ensured by the force sensor recordings provided by CathROB.

For each procedure, both fluoroscopy time and 3D map construction time were assessed. At the end of the procedure, the animal was sacrificed and the chest was opened for heart excision. The cardiac chambers were visually inspected to observe any damage to endocardial structures and to verify the effectiveness of ablation lesions. Mean ± standard deviation was used to present the collected experimental data for both in vitro and in vivo evaluations.

## 3. Results

### 3.1. In Vitro Results

The map of LA chamber in the silicone model was remotely reconstructed using 35 CARTO points. The total time for map reconstruction was 34 minutes and fluoroscopy time was 18 minutes.

**Figure 10:**
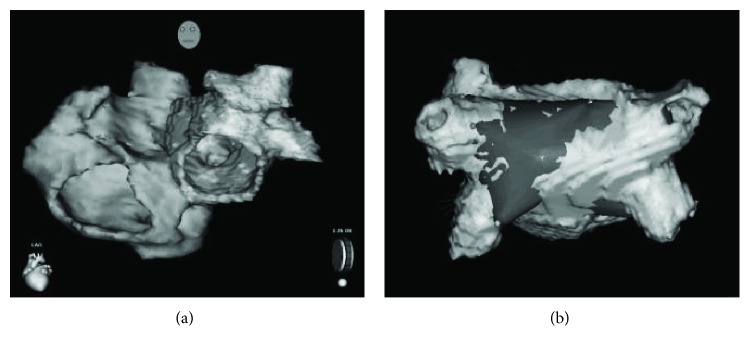
(a) CT-derived reconstruction of the LA chamber of the mock silicone model; (b) the reconstructed CARTO map (dark grey area) merged to CT reconstruction.

Good accuracy was achieved for the remotely reconstructed map, since the overall deviation index measured by the CARTOMERGE software was 1.37 ± 0.98 mm (Figure 10).

The mean repositioning errors estimated for the repeated automatic repositioning of the catheter to target sites in the proximity of pulmonary veins (PV) were 1.5 ± 0.6 mm for the left-side targets (left anterior and posterior PV) and 1.7 ± 0.5 mm for the right-side targets (right anterior and posterior PV).

The force-sensing and feedback mechanism were effective in avoiding any damage or perforation of the silicone model, since the robotic hand automatically stops when the “stop threshold” was exceeded.

### 3.2. In Vivo Results

The mean set-up time for CathROB installation in the four experimental sessions was 5 ± 3 min.

For all the experiments, remote RA mapping and RF ablation were achieved safely, that is, without causing any injury for cardiac tissues or inducing any alteration in cardiac rhythm in the animals. On average, the RA maps were reconstructed with 63 ± 8 CARTO maps points. The mean total map reconstruction time was 36 ± 7 min and fluoroscopy time was 15 ± 3 min (Table 3).

**Figure 11:**
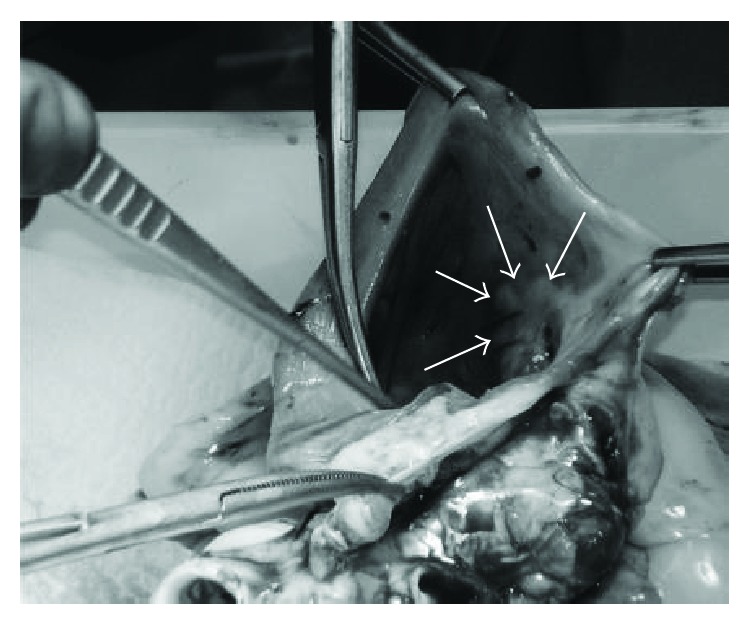
Visible RF lesions (indicated by the white arrows) obtained during in vivo tests, in the region of RA isthmus.

These values were comparable to those of manually navigated catheters [30]. Using the CathROB system, the operator successfully positioned the ablation catheter to all the predesigned endocardial target sites for ablation (RA isthmus and RA posterior wall). All three experienced electrophysiologists who were directly involved in the execution of the experiments clearly identified, via visual inspection of the excised heart, the achieved effective RF lesions, and they confirmed that these were comparable with the one they typically obtain when manually maneuvering standard ablation catheters (Figure 11).

There were no adverse events observed, including absence of cardiac perforation, indicative of the safety profile of the CathROB system. The switching between remote control and standard manual handling of the catheter was performed quickly and easily without breaking sterility, as well as the reattachment of the catheter on the robotic hand to switch back to remote navigation.

## 4. Discussion

In this paper we present CathROB, a highly compact and versatile robotic device that allows 3-DOF remote manipulation of conventional tip-steerable EP catheters, without the need for the operator to be in the X-ray field.

The distinctive features of the presented CathROB system include an extremely compact and lightweight design, the fast device installation and ease of operation, the inherently safe design, and the automatic navigation functionalities that may have potential of reducing the overall procedural time (Table 4).

The CathROB safety profile is ensured by preservation of the mechanical properties of a standard EP catheter, as well as by the included force-sensing technology. CathROB also ensures the minimization of vascular complications since the remotely controlled catheter enters the vasculature through a standard sheath introducer in the groin.

Having perception of the contact between the catheter tip and the endocardium is an important feature in transcatheter ablation procedures, since contact is a requirement for performing effective lesions. For this study, we determined the alarm/stop thresholds for the force sensor, for an ovine heart. Surely, when using CathROB for remote catheter navigation in human hearts, we would need to repeat this threshold determination. Systems like the Amigo robotic arm, which lacks a reactive force feedback, require the use of specialized catheters with contact force-sensing capabilities [31] to obtain perception on catheter-endocardium contact, and this probably increases the overall cost of the procedure.

The interesting feature of the CathROB force-sensing mechanism is the haptic feedback provided to the operator by transmitting back to his hand a vibrational force via the control mock handle, when alarm or stop threshold is exceeded. This feature has been particularly appreciated by the physicians who experimented the CathROB system.

Unlike other proposed remote catheter navigation systems [4–6, 10–15], CathROB is not constrained to the use of dedicated catheters/sheaths or to the need for a specialized room. Conversely, being an open platform adaptable to any commercially available standard EP catheter, it may represent a less costly alternative that does not limit the physician's choice for their standard sheaths and catheters.

Similar to the Amigo system [16–18], the intuitive CathROB command interface that replicates the manual maneuvering of a standard catheter handle has been designed to take advantage of the operator's acquired dexterity and ultimately to shorten the learning curve. Compared to the Amigo system, CathROB offers the additional features of automatic catheter navigation and repositioning to pre-explored endocardial targets. This may be advantageous for complex EP procedures that may require repeated repositioning of the catheter to endocardial target sites and has potential to reduce the total procedural time and the fluoroscopy time for the patient [26].

To date, most of the available robotic solutions are addressed toward complex ablation procedures, such as atrial fibrillation. However, considering that standard arrhythmias, such as supraventricular tachycardia, ventricular tachycardia, and atrial flutter, are still occupying about a half of the cath lab volumes [32], it could be advisable to address robotic platforms also to serve these arrhythmia categories. The CathROB, being a compact and easy-to-install system, has the potential to be a versatile device that can be used for both complex ablation procedures in atrial fibrillation and simpler ablation treatments for more standard arrhythmias. Moreover, CathROB has potential for cost savings: if we assume that a mean set-up time for a commercial robotic catheter navigation system like Magellan or Niobe is about 30 minutes [7–9, 15], we could save about 25 minutes of cath lab activity, for each procedure when using CathROB. This, in a cath lab performing about 1000 procedures annually, corresponds to savings of about 417 hours of activity, per year. Considering the indicative cost of $48 per hour of cath lab activity reported by Professor Adhir Shroff [35], CathROB could allow cost savings of about $20,000 per year.

In our animal evaluation, we limited the use of CathROB to remotely navigate the catheter inside the RA, while in the mock silicone heart, we maneuvered the catheter also inside the LA, as needed for complex ablation in atrial fibrillation. For LA navigation, we preliminarily performed the transseptal passage by manual maneuvering. In order to accomplish remotely all the procedural steps needed for catheter ablation in atrial fibrillation, also the maneuvering of sheaths required for transseptal puncture should be performed remotely through a robotic hand, as well as the navigation of circumferential Lasso mapping catheters used to confirm the achievement of pulmonary vein isolation after RF ablation. Recently, a remote Lasso catheter manipulation system was developed as an additional feature of the Niobe magnetic [33]. However, the major limitation remains, the encumbrance of the overall Niobe equipment in the cath lab. On the other hand, the extremely compact CathROB design may allow installing two or more robotic hands in the same cath lab to remotely manipulate more than one catheter or device simultaneously.

At this stage of development, CathROB has been mainly addressed to remotely navigate EP catheters. However, it could be easily extended in the future to be used to remotely navigate endovascular catheters for percutaneous coronary intervention or peripheral interventions. Although the presented CathROB must undergo further preclinical and clinical studies to validate its efficacy, the initial results we presented are very promising.

## 5. Conclusions

We introduced CathROB, a new robotically driven system for remote catheter navigation.

The compact and versatile design and the fast set-up operation, with the additional features of a safe automatic catheter navigation, make CathROB very interesting and attractive for its practical use in cath labs.

This study demonstrated the feasibility and safety of the presented CathROB system for remote catheter navigation and cardiac mapping, as well as for RF ablation in vivo.

## Supplementary Material

Figure S1. Elasticity of APPJ-treated RBCs measured by atomic force microscopy. Figure S2. Raman spectroscopy of RBC and RBC lipid membrane after Air and N2 APPJ treatment and their PCA analysis.

## Figures and Tables

**Table 1:** Commercially available remote catheter navigation systems.

System (company)	Technology	Intended use	Features	Main limitations
Niobe (Stereotaxis)	Magnetic	RF ablation	Use of dedicated large magnets; remote navigation of a dedicated magnetic catheter with a soft tip	Need for a specially designed catheter and a room dedicated to the magnets; encumbrance and complexity of the overall system set-up

Sensei/Magellan (Hansen Medical)	Electromechanical	RF ablation/vascular procedures	Use of dedicated steerable sheaths for remote catheter control	Need for a dedicated custom-designed sheath; risk of mechanical complications due to the rigidity of the sheath

Amigo (Catheter Precision)	Electromechanical	RF ablation	Remote manipulation of standard tip steerable EP catheters; remote controller that mimics the handle of standard EP catheters	Encumbrance of the system

CorPath (Corindus)	Electromechanical	Percutaneous coronary interventions	Remote manipulation of standard guidewires and balloon/stent catheters	Need for a dedicated single-use cassette to maneuver the catheters

**Table 2:** Description of GUI functionalities.

Block	GUI component	Function description
(1) Start/stop commands	START softkey	To start the automatic CathROB movements for system set-up (no catheter mounted on)
	STOP softkey	To stop all CathROB movements
	CATHETER slider	To select the catheter model to be remotely controlled by CathROB

(2) CathROB status	E-STOP led	To indicate an emergency stop
	MOTOR CURRENT led	To indicate an abnormal current absorption by motors
	ROB On led	To indicate that CathROB is in on-state
	ENABLE led	To indicate that the operator is using the command interface
	CALIBRATING led	To indicate that calibration procedure for the force sensor is in progress
	OK led	To indicate that calibration was successful
	NAVIGATING led	To indicate that the user is performing remote catheter navigation
	MEMO led	To indicate that the user is saving endocardial target positions via the command interface
	REPOSITIONING led	To indicate that the system is performing the automatic catheter repositioning to memorized targets
	L, R, S led	To indicate that repositioning has been completed for longitudinal (L), rotational (R), and tip-steering (S) movements

(3) Targets of interest	Home/T1/T2/T3/T4 indicators	To display the coordinates of the targets saved during navigation via the command interface
	LSPV, RSPV, LIPV, RIPV softkeys	To load in the system memory four predefined endocardial sites (corresponding to the pulmonary veins)

(4) CathROB display	ADVANCE/WITHDRAWAL; CW/CCW; BEND/STRAIGTHEN indicators	To display the real-time CathROB movements along each DOF
	MIN, MEDIUM, MAX selectors	To change motor velocities in each DOF
	S/H led	To indicate when the actuators reach software/hardware limit switches

(5) Force sensing	FORCE graph	To display the force signal measured by the force sensor
	SAFETY ALARM led	To indicate that force exceeds the set alarm threshold
	SAFETY STOP led	To indicate that force exceeds the set stop threshold

**Table 3:** Results for remote RA mapping and RF ablation obtained during in vivo animal experiments.

	Map time (min)	Fluoroscopy time (min)	CARTO points	Target ablation sites	RF energy (W)	Ablation success
Case						
1	35	13	53	RA isthmus	30	Yes
				RA pos. wall	35	Yes
2	44	19	65	RA isthmus	30	Yes
				RA post. wall	25	Yes
3	28	15	60	RA isthmus	35	Yes
				RA pos. wall	40	Yes
4	38	12	92	RA isthmus	25	Yes
				RA pos. wall	25	Yes
Mean	36	15	63			
DS	7	3	8			

**Table 4:** Comparison of CathROB with commercially available remote catheter navigation systems.

Feature	Remote catheter navigation systems				
	CathROB	Niobe	Sensei/Magellan	Amigo	CorPath
Compact design and fast installation	Yes	No	No	No	Yes [20]
Use of standard catheters/sheaths	Yes	No	No	Yes [16]	No
Intuitive command interface	Yes	No	No	Yes [18]	No
Force-sensing technology	Yes	No	Yes [34]	No	No
Automatic catheter navigation	Yes	Yes [6]	No	No	No
